# Supplementary material for: A high-throughput multiparameter screen for accelerated development and optimization of soluble genetically encoded fluorescent biosensors
Source: Nat Commun. 2022 May 25;13:2919. doi: 10.1038/s41467-022-30685-x (PMC9133083; doi:10.1038/s41467-022-30685-x)
Supplement: Supplementary file 1 — Supplementary Information [file 41467_2022_30685_MOESM1_ESM.pdf]

**SUPPLEMENTARY INFORMATION**

# A high-throughput multiparameter screen for accelerated development and optimization of soluble genetically encoded fluorescent biosensors

Dorothy Koveal, Paul C. Rosen, Dylan J. Meyer, Carlos Manlio Díaz-García, Yongcheng Wang, Li-Heng Cai, Peter J. Chou, David A. Weitz, and Gary Yellen

CONTENTS

Supplementary Fig. 1 Optimization of IVTT reaction in droplets

Supplementary Fig. 2 Custom microfluidic devices used to generate biosensor library GSBs

Supplementary Fig. 3 Recovery of biosensor DNA from single GSBs

Supplementary Fig. 4 TlpC-TQ library screen yields a pH-resistant lactate biosensor

Supplementary Fig. 5 Normalized excitation and emission spectra of LiLac for lactate-bound and -unbound states

Supplementary Fig. 6 Fluorescence lifetime readout of LiLac versus Laconic in HEK293T cells

Supplementary Fig. 7 Analysis of LiLac intensity responses in HEK293T cells

Supplementary Table 1 Photophysical properties of LiLac

Supplementary Note 1 DNA and protein sequences for LiLac

Supplementary References

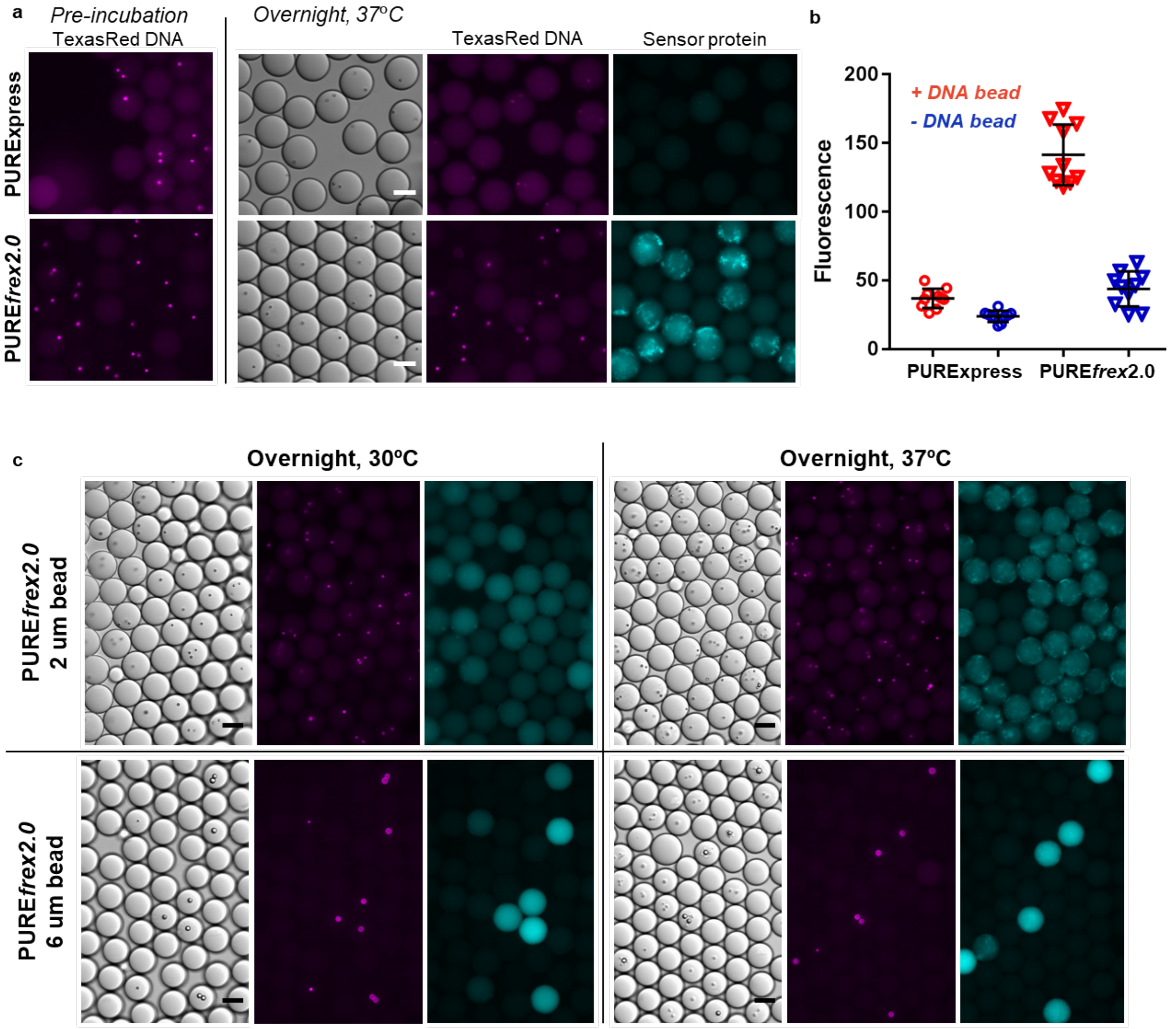


**Supplementary Figure 1. Optimization of IVTT reaction in droplets.**

a) Expression of single biosensor clones in microfluidic droplets containing purified reagents for *in vitro* transcription/translation (IVTT). 5'-TexasRed/5'-DualBiotin-tagged DNA immobilized on streptavidin beads (2 µm diameter) were encapsulated in droplets containing purified IVTT reagents (PURExpress or PUREfrex2.0) and incubated overnight at 37ºC. While PUREfrex2.0 droplets yielded mature biosensor protein, PURExpress droplets did not. The reason for this difference was undetermined, but may be related to the observed loss of red fluorescence from the DNA-capture beads following overnight incubation in PURExpress reagents.

b) Quantification of the average green biosensor fluorescence intensity within individual droplets imaged in (a), n = 10 droplets for each, mean ± SD.

c) Optimization of DNA-capture bead size and copy number. Streptavidin beads were loaded with TexasRed/Biotin-tagged DNA (~10–20,000 copies/bead for saturated 2 µm diameter beads, or ~100,000 copies/bead for sparsely loaded 6 µm diameter beads), encapsulated in PUREfrex2.0 droplets, and incubated overnight at 30 or 37ºC. Larger beads carrying a higher copy number yielded higher expression levels of mature biosensor protein.

All scale bars represent 25 µm.

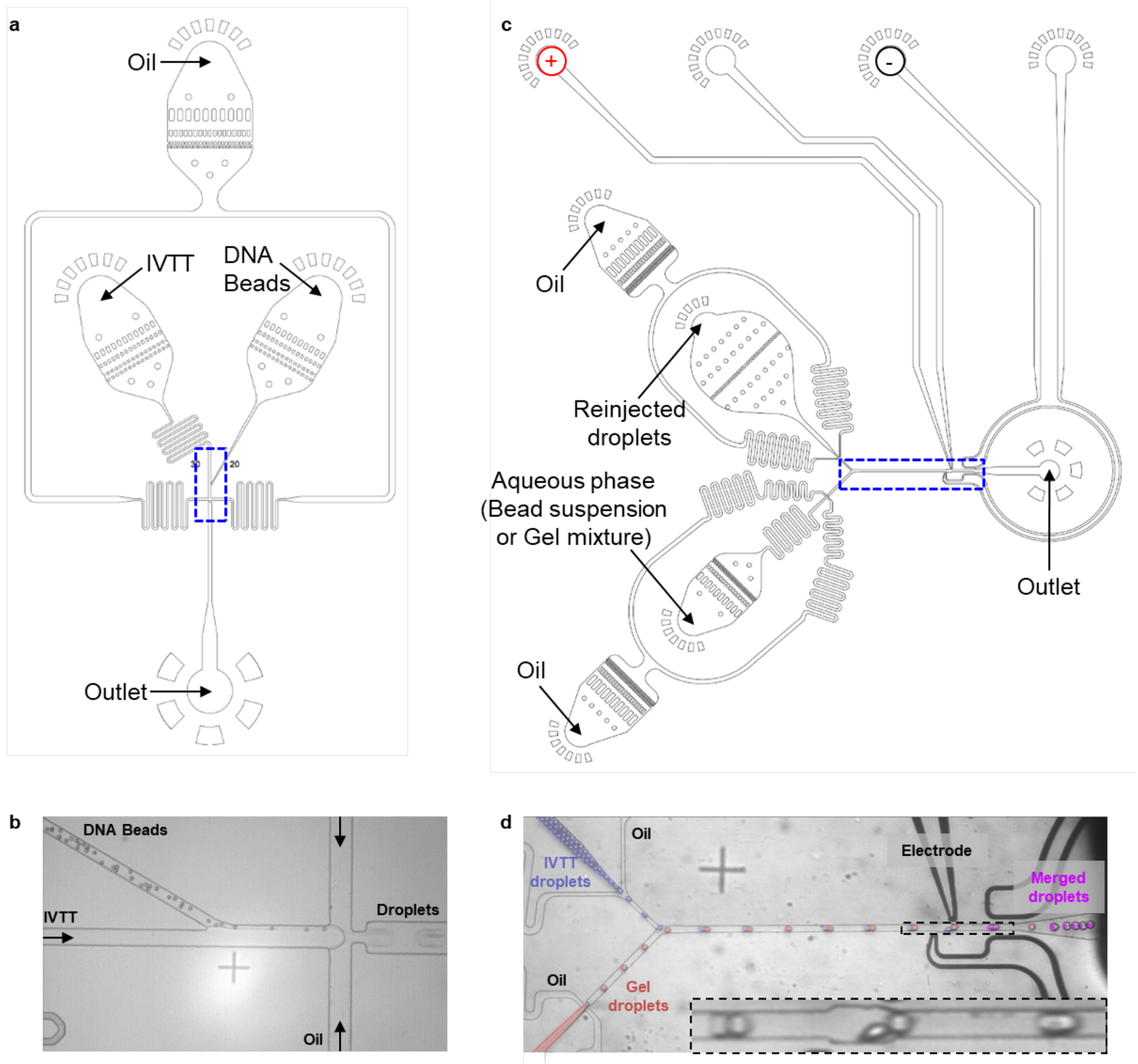


**Supplementary Figure 2. Custom microfluidic devices used to generate biosensor library GSBs**

a) Schematic of a two-stream co-flow droplet generator device used to introduce DNA beads into IVTT reagents immediately prior to isolation in droplets. Inlets for droplet stabilization oil, IVTT reagents, and the DNA bead suspension are indicated, as well as the emulsion outlet.

b) A snapshot of a typical experimental run. The field of view matches the blue boxed region in (a) rotated by 90°.

c) Schematic of the electrocoalescence device used to merge paired droplets. The same device is used for merging PCR droplets with streptavidin beads (Fig 2b) or for merging IVTT droplets with gel droplets (Fig 2e). Positive (red circle) and ground (black circle) electrodes are indicated.

d) Snapshot of an electrocoalescence experiment merging IVTT droplets with gel droplets, the penultimate step in generating GSBs. The field of view matches the blue boxed region in (c), and the inset shows a magnified view at the point of droplet fusion.

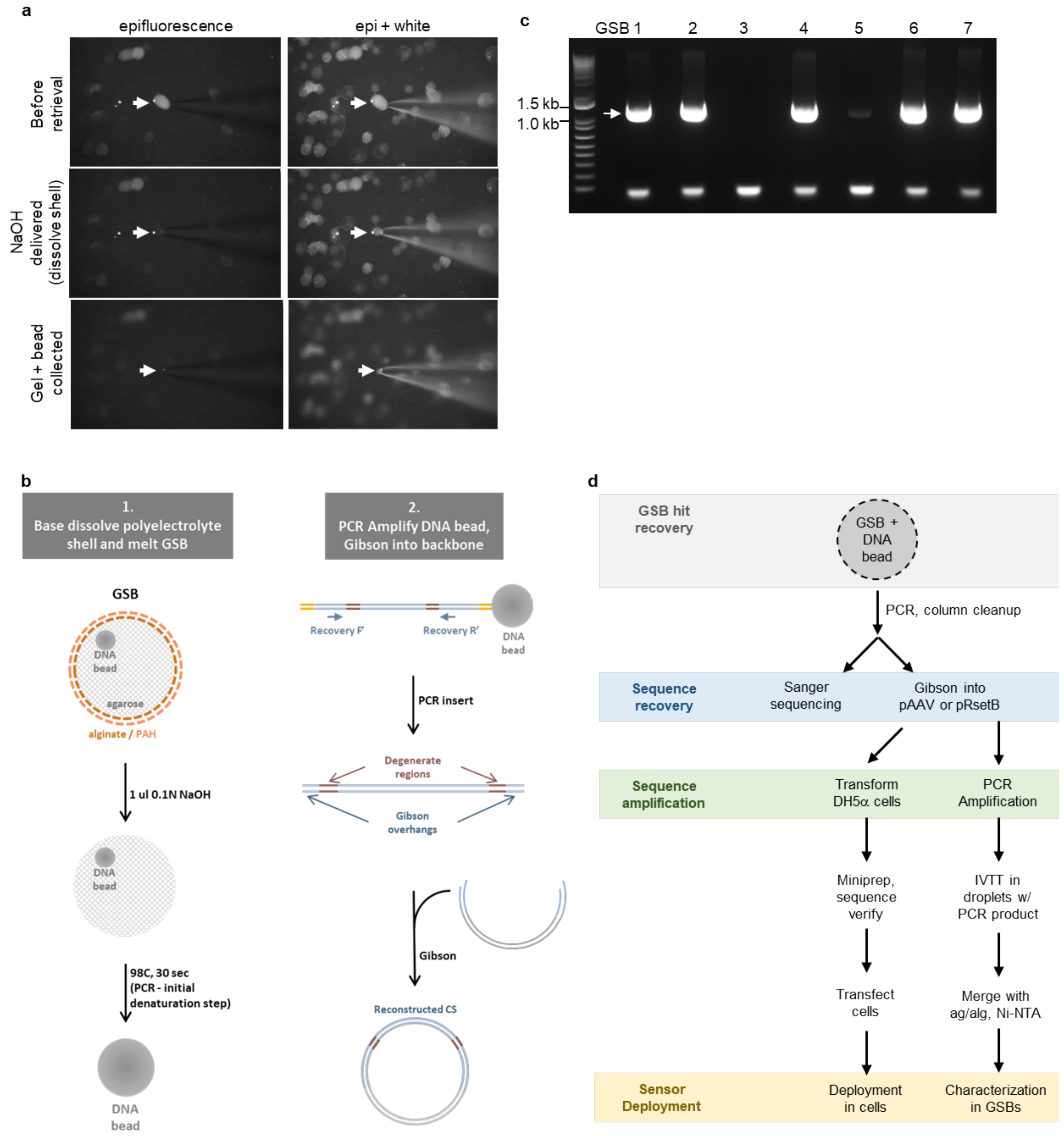


**Supplementary Figure 3. Recovery of biosensor DNA from single GSBs.**

a) Retrieval of a single GSB following library screening. A glass micropipette with a 15–30 µm tip diameter is loaded with 0.1N NaOH and placed next to the target GSB, indicated by the white arrow (top panels). The polyelectrolyte shell is base dissolved (middle panels), which facilitates manipulation of the gel and provides access to PCR reagents in downstream steps. Breaching the shell also releases the encapsulated biosensor protein, which can be seen as a loss of fluorescence in the green epifluorescence channel. However, the DNA bead (small bright puncta) remains embedded in the agarose gel. Both the agarose gel and the DNA bead are aspirated into the microcapillary tip (bottom panels), removed from the perfusion chamber and delivered into a tube for DNA recovery by PCR.

b) Workflow for DNA recovery from GSBs. First, the GSB shell is melted with 0.1 N NaOH, yielding a DNA bead embedded in agarose gel, which is subjected to PCR. The agarose gel is melted during the first denaturation step in PCR, releasing the DNA bead. Recovery primers that anneal to the biosensor gene just outside of the degenerate linker regions are used to amplify a portion of the biosensor, which is later recombined with the rest of the staging plasmid via Gibson assembly to regenerate the full coding sequence (CS).

c) PCR amplified DNA from each of seven independently recovered GSBs, the white arrow indicating the expected band size. DNA sequences were successfully recovered from six of the seven samples.

d) Workflow of biosensor hit recovery, characterization and deployment.

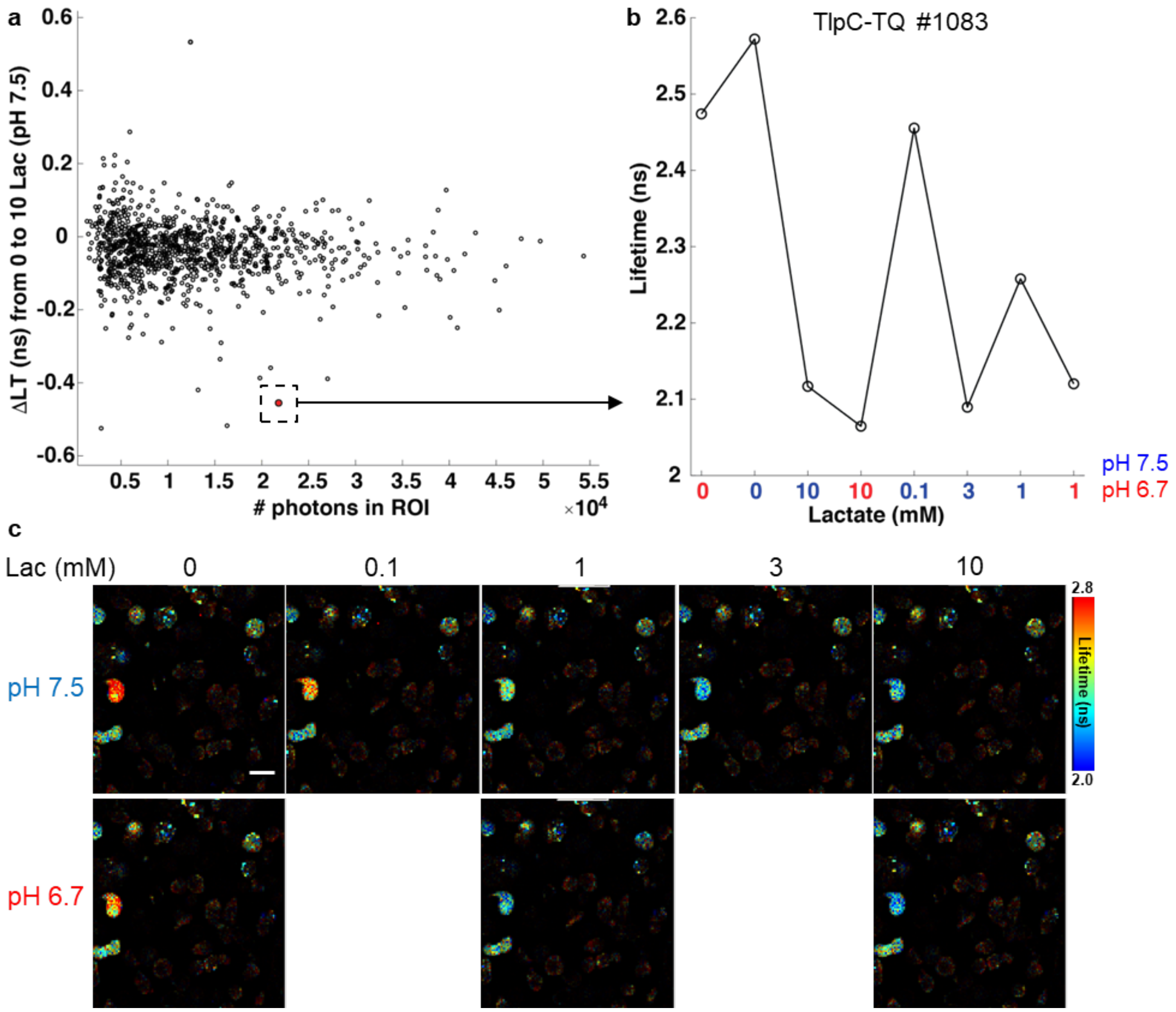


**Supplementary Figure 4. TlpC-TQ library screen yields a pH-resistant lactate biosensor.**

a) Results from the initial screen of the TlpC-TQ lactate biosensor library. Average photon counts within each ROI are plotted against the maximum change in lifetime between 0 and 10 mM lactate at pH 7.5, and each data point represents a single biosensor variant within a GSB from a total of 1,411 screened. The red data point and dotted box indicate TlpC-TQ #1083, which was highly resistant to changes in pH.

b) Lifetime values for the TlpC-TQ #1083 variant at the indicated lactate concentrations and pH's, plotted in the order in which the data were collected during the screen.

c) Filmstrip of the lifetime images used to generate the data plotted in (b). Scale bar indicates 100 μm.

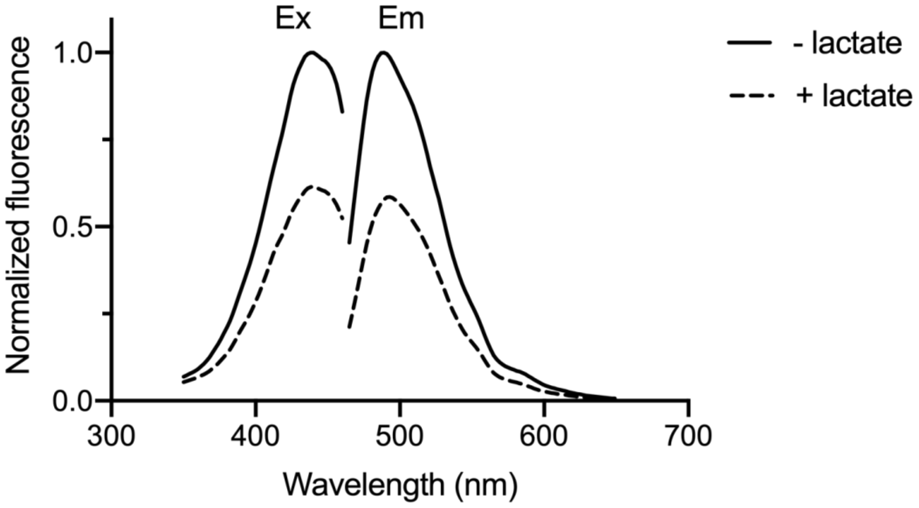


**Supplementary Figure 5. Normalized excitation and emission spectra of LiLac for lactate-bound and -unbound states.** Excitation and emission spectra were measured for purified LiLac protein in the lactate-unbound (Apo, solid line) and lactate-bound (Sat, dashed line) states, in technical quadruplicates, averaged and normalized to the peak intensity in the Apo condition. For excitation spectra, emission was measured at 500 nm; for emission spectra, excitation was at 425 nm.

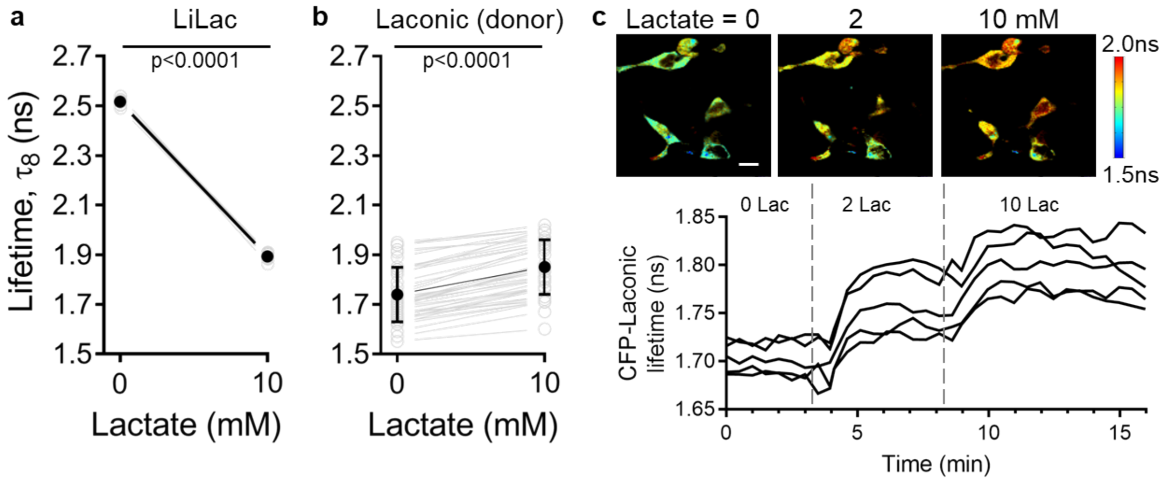


**Supplementary Figure 6. Fluorescence lifetime readout of LiLac versus Laconic in HEK293T cells.**

a) HEK293T cells expressing LiLac display a lifetime change ($\Delta\tau_8$) of -0.62 ± 0.04 ns (mean ± SD, $N_{cells}$=15 from one experiment) from 0 to 10 mM lactate.

b) HEK293T cells expressing Laconic display a donor lifetime change of 0.11 ± 0.04 ns (mean ± SD, $N_{cells}$=45 from six independent experiments), with variable lifetime values across cells. Signal-to-noise values for LiLac and Laconic were computed from these data as described in the methods, yielding $LiLac_{SNR}$ ~ 25 and $Laconic_{donor\ lifetime,SNR}$ < 1.

c) The representative filmstrip and single cell traces show that the lifetime of the donor species in Laconic varies across cells, even though it does increase in response to the application of external lactate. HEK293T cells were transfected with the Laconic/pcDNA3.1(-) plasmid.[1] The transfection protocol, recording conditions, data analysis, and 2p-FLIM imaging settings for Laconic CFP (donor) emission have been previously described.[2,3] Experiments were performed in a glucose-free bath solution, with 0.1 mM of the glycolytic inhibitor iodoacetate for the Laconic recordings. Scale bar indicates 15 µm.

The data in a and in b were compared using a Wilcoxon matched pairs test (two-sided), yielding the indicated p-values and test statistic, W of 120 for LiLac and 1035 for Laconic (donor). Individual data points are shown in gray, and mean ± SD are plotted in black. Some error bars are occluded by the data point.

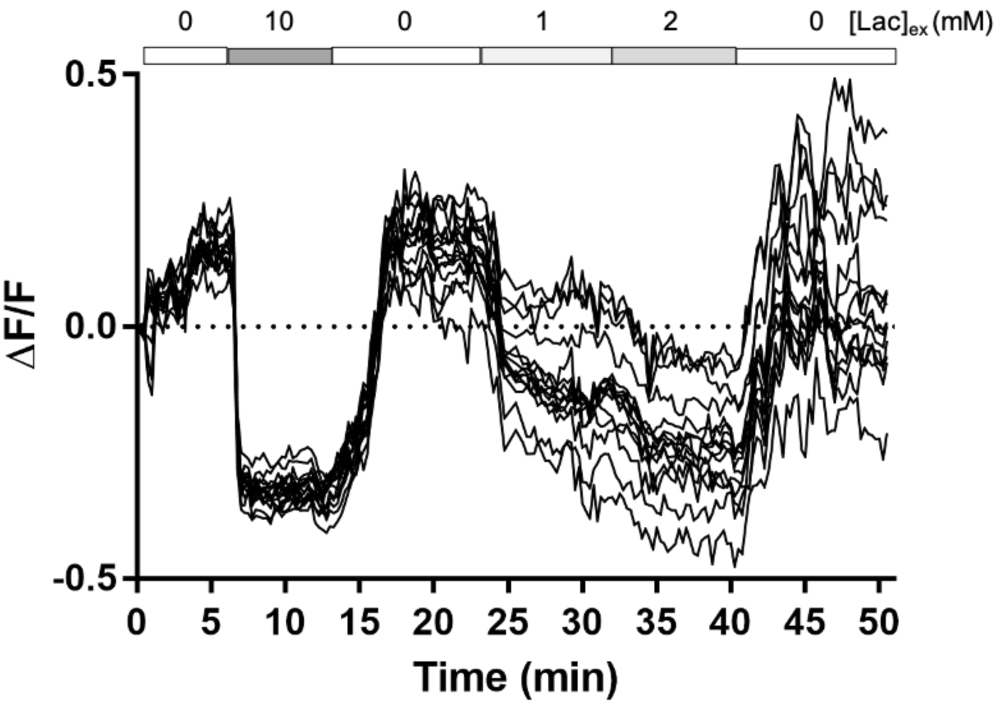


**Supplementary Figure 7. Analysis of LiLac intensity responses in HEK293T cells.**

Corresponding normalized intensity traces (ΔF/F). The error associated with ΔF/F measurements increases over the course of an experiment ($\Delta F/F_{4min}$ = 0.15 ± 0.03, $\Delta F/F_{19min}$ = 0.18 ± 0.05, $\Delta F/F_{49min}$ = 0.06 ± 0.16, mean ± SD calculated over a 1 minute window for each timepoint), possibly due to photobleaching effects, while the fluorescence lifetime is unaffected. However, if one intends to quantify intensity changes, imaging parameters can be adjusted to minimize the effects of photobleaching, and an inert fluorescence tag (e.g. mCherry) can be covalently attached to LiLac as a means of normalizing for protein expression levels.[4,5]

**Supplementary Table 1. Photophysical properties of LiLac**

| | $\lambda_{ex}$ (nm) | $\lambda_{em}$ (nm) | QY | $\varepsilon$ ($10^3$ $M^{-1}cm^{-1}$) |
|---|---|---|---|---|
| Apo | 438 | 489 | 0.87 | 31.0 |
| Sat | 440 | 494 | 0.56 | 28.6 |

Spectral properties $\lambda_{ex}$: excitation maximum, $\lambda_{em}$: emission maximum, QY: quantum yield relative to acridine orange, $\varepsilon$: extinction coefficient determined at 440 nm. Apo and Sat are in the presence of 0 or 100 mM lactate, respectively.

**Supplementary Note 1. DNA and protein sequences for LiLac**

**LiLac Protein sequence**

MKHHHHHHHGVSKGEELFTGVVPILVELDGDVNGHKFSVSGEGEGDATYGKLTLKFICTTGKLP
VPWPTLVTTLSWGVQCFARYPDHMKQHDFFKSAMPEGYVQERTIFFKDDGNYKTRAEVKFEGDT
LVNRIELKGIDFKEDGNILGHKLEYNSSGRTGIDPFTESVLQSQATELLQKKAQLVSFKIQGIM
KRIFMGANTLEKFLSDENSAINDTLKRRMLSEFLLANPHVLLVSAIYTNNNERVITAMSMDSKI
AYPNTTLNENMTNQIRSLKSITHSDPYYKEVNGDKIYGMDITLPLMGKNQNAIGALNFFLNIDA
FYTDVVGKKKSNTFLMGKDGRLLINPNREIQDKILSAINPDRRVAKAVEYYNQNEAGTLSYHSL
SGNTETFLAIQPFDFFEEKGNNGNHWRWAIGKYVNKSLVFSSHSNVYITADKQKNGIKANFKIR
HNIEDGGVQLADHYQQNTPIGDGPVLLPDNHYLSTQSKLSKDPNEKRDHMVLLEFVTAAGITLG
MDELYQ

mTurquoise2
Glycine present only in pAAV-CAG-LiLac construct
TlpC
linkers

**LiLac DNA sequence**

ATGAAACATCACCATCATCATCATCATGGAGTGAGCAAGGGCGAGGAGCTGTTCACCGGGGTGG
TGCCCATCCTGGTCGAGCTGGACGGCGACGTAAACGGCCACAAGTTCAGCGTGTCCGGCGAGGG
CGAGGGCGATGCCACCTACGGCAAGCTGACCCTGAAGTTCATCTGCACCACCGGCAAGCTGCCC
GTGCCCTGGCCCACCCTCGTGACCACCCTGTCCTGGGGCGTGCAGTGCTTCGCCCGCTACCCCG
ACCACATGAAGCAGCACGACTTCTTCAAGTCCGCCATGCCCGAAGGCTACGTCCAGGAGCGCAC
CATCTTCTTCAAGGACGACGGCAACTACAAGACCCGCGCCGAGGTGAAGTTCGAGGGCGACACC
CTGGTGAACCGCATCGAGCTGAAGGGCATCGACTTCAAGGAGGACGGCAACATCCTGGGGCACA
AGCTGGAGTACAACTCCTCCGGCCGCACCGGCATTGACCCCTTTACTGAAAGCGTGTTGCAAAG
CCAAGCCACAGAATTGCTGCAAAAAAAAGCTCAGTTAGTCAGTTTTAAAATTCAAGGCATTATG
AAGCGCATTTTTATGGGCGCTAACACCCTTGAAAAATTTTTAAGCGATGAAAATAGCGCTATCA
ACGACACCCTCAAACGCCGCATGCTCTCTGAGTTTTTGTTAGCAAACCCTCATGTGTTATTGGT
TAGCGCGATTTATACGAATAATAATGAACGTGTCATCACTGCCATGAGCATGGATTCAAAAATC
GCCTACCCTAATACCACGCTCAATGAAAACATGACCAATCAAATCCGTTCGCTCAAAAGTATTA
CCCATTCAGATCCCTATTATAAAGAGGTTAATGGCGATAAAATCTATGGCATGGATATTACCCT
CCCCCTTATGGGTAAGAATCAAAATGCTATTGGCGCGCTGAATTTCTTTTTAAACATTGACGCT
TTTTATACCGATGTGGTAGGCAAGAAAAAGAGCAACACCTTTTTAATGGGGAAAGACGGCCGTC
TTTTAATCAACCCTAATCGTGAGATCCAAGATAAGATTTTAAGCGCTATCAATCCAGATCGCCG
TGTCGCTAAAGCTGTGGAGTATTACAATCAAAACGAAGCGGGCACTTTGAGCTACCATTCATTG
AGCGGGAATACAGAAACCTTTTTAGCCATTCAGCCCTTTGATTTTTTTGAAGAAAAAGGGAATA
ACGGCAATCATTGGCGTTGGGCAATTGGGAAATATGTCAATAAATCTTTAGTCTTTTCCAGCCA
CAGCAACGTCTATATCACCGCCGACAAGCAGAAGAACGGCATCAAGGCCAACTTCAAGATCCGC
CACAACATCGAGGACGGCGGCGTGCAGCTCGCCGACCACTACCAGCAGAACACCCCCATCGGCG
ACGGCCCCGTGCTGCTGCCCGACAACCACTACCTGAGCACCCAGTCCAAGCTGAGCAAAGACCC
CAACGAGAAGCGCGATCACATGGTCCTACTGGAGTTCGTGACCGCCGCCGGGATCACTCTCGGC
ATGGACGAGCTGTACCAG

**TlpC-TQ Library DNA sequence (as immobilized on DNA beads)**

CGCGTTGGCCGATTCATTAATGCAGGATCTCGATCCCGCGAAATTAATACGACTCACTATAGGG
AGACCACAACGGTTTCCCTCTAGAAATAATTTTGTTTAACTTTAAGAAGGAGATATACATATGA
AACATCACCATCATCATCATCATGTGAGCAAGGGCGAGGAGCTGTTCACCGGGGTGGTGCCCAT

CCTGGTCGAGCTGGACGGCGACGTAAACGGCCACAAGTTCAGCGTGTCCGGCGAGGGCGAGGGC
GATGCCACCTACGGCAAGCTGACCCTGAAGTTCATCTGCACCACCGGCAAGCTGCCCGTGCCCT
GGCCCACCCTCGTGACCACCCTGTCCTGGGGCGTGCAGTGCTTCGCCCGCTACCCCGACCACAT
GAAGCAGCACGACTTCTTCAAGTCCGCCATGCCCGAAGGCTACGTCCAGGAGCGCACCATCTTC
TTCAAGGACGACGGCAACTACAAGACCCGCGCCGAGGTGAAGTTCGAGGGCGACACCCTGGTGA
ACCGCATCGAGCTGAAGGGCATCGACTTCAAGGAGGACGGCAACATCCTGGGGCACAAGCTGGA
GTACAAC**TYCWMCGSCSRCRYC**GGCATTGACCCCTTTACTGAAAGCGTGTTGCAAAGCCAAGCC
ACAGAATTGCTGCAAAAAAAAGCTCAGTTAGTCAGTTTTAAAATTCAAGGCATTATGAAGCGCA
TTTTTATGGGCGCTAACACCCTTGAAAAATTTTTAAGCGATGAAAATAGCGCTATCAACGACAC
CCTCAAACGCCGCATGCTCTCTGAGTTTTTGTTAGCAAACCCTCATGTGTTATTGGTTAGCGCG
ATTTATACGAATAATAATGAACGTGTCATCACTGCCATGAGCATGGATTCAAAAATCGCCTACC
CTAATACCACGCTCAATGAAAACATGACCAATCAAATCCGTTCGCTCAAAAGTATTACCCATTC
AGATCCCTATTATAAAGAGGTTAATGGCGATAAAATCTATGGCATGGATATTACCCTCCCCCTT
ATGGGTAAGAATCAAAATGCTATTGGCGCGCTGAATTTCTTTTTAAACATTGACGCTTTTTATA
CCGATGTGGTAGGCAAGAAAAAGAGCAACACCTTTTTAATGGGGAAAGACGGCCGTCTTTTAAT
CAACCCTAATCGTGAGATCCAAGATAAGATTTTAAGCGCTATCAATCCAGATCGCCGTGTCGCT
AAAGCTGTGGAGTATTACAATCAAAACGAAGCGGGCACTTTGAGCTACCATTCATTGAGCGGGA
ATACAGAAACCTTTTTAGCCATTCAGCCCTTTGATTTTTTTGAAGAAAAAGGGAATAACGGCAA
TCATTGGCGTTGGGCAATTGGGAAATATGTCAATAAATCTTTAGTCTTT**TYCMRCSMCHRC**AAC
GTCTATATCACCGCCGACAAGCAGAAGAACGGCATCAAGGCCAACTTCAAGATCCGCCACAACA
TCGAGGACGGCGGCGTGCAGCTCGCCGACCACTACCAGCAGAACACCCCCATCGGCGACGGCCC
CGTGCTGCTGCCCGACAACCACTACCTGAGCACCCAGTCCAAGCTGAGCAAAGACCCCAACGAG
AAGCGCGATCACATGGTCCTACTGGAGTTCGTGACCGCCGCCGGGATCACTCTCGGCATGGACG
AGCTGTACCAGTGATTTCCGGACTAGCATAACCCCTTGGGGCCTCTAAACGGGTCTTGAGGGGT
TTTTTGCTGAGCTCCGAGGGGCCCAAGCTGTAGTGGCCATCGCCCTGATAGACGGTTTTTCGC
CCTTTGACGTTGGAGTCCACGTTCTTTAATAGTGGACTCTTGTTCCAAACTGGAACAACACTCA
ACCCTATCTCGGTCTATTCTTTTGATTTATAAGGGATTTTGCCGATTTCGGCCTATTGGTTAAA
AAATGAGCTGATTTAACAAAAATTTAACGCGAATTTTAACAAAATATTAACGCTTACAATTTAG
GTGGCACTTTTCGGGGAAATGTGCGCGGAACCCCTATTTGTTTATTTTTCTAAATACATTCAAA
TATGTATCCGCTCATGAGACAATAACCCTGATAAATGCTTC

TexasRed-Forward primer annealing
DualBiotin-Reverse primer annealing
**Degenerate linker regions**

**Supplementary References**


1. San Martín, A. *et al.* A genetically encoded FRET lactate sensor and its use to detect the Warburg effect in single cancer cells. *PLoS One* **8**, e57712 (2013).
2. Díaz-García, C. M. *et al.* Neuronal stimulation triggers neuronal glycolysis and not lactate uptake. *Cell Metab* **26**, 361-374.e4 (2017).
3. Díaz-García, C. M. *et al.* Quantitative in vivo imaging of neuronal glucose concentrations with a genetically encoded fluorescence lifetime sensor. *J Neurosci Res* **97**, 946–960 (2019).
4. Tantama, M., Martínez-François, J. R., Mongeon, R. & Yellen, G. Imaging energy status in live cells with a fluorescent biosensor of the intracellular ATP-to-ADP ratio. *Nat Commun* **4**, 2550 (2013).
5. Hung, Y. P., Albeck, J. G., Tantama, M. & Yellen, G. Imaging cytosolic NADH-$NAD^+$ redox state with a genetically encoded fluorescent biosensor. *Cell Metab* **14**, 545–554 (2011).
