# Supplementary material for: A high-throughput multiparameter screen for accelerated development and optimization of soluble genetically encoded fluorescent biosensors
Source: Nat Commun. 2022 May 25;13:2919. doi: 10.1038/s41467-022-30685-x (PMC9133083; doi:10.1038/s41467-022-30685-x)
Supplement: Supplementary file 3 — Reporting Summary [file 41467_2022_30685_MOESM3_ESM.pdf]

nature portfolio

Corresponding author(s): Gary Yellen
Last updated by author(s): May 2, 2022

Reporting Summary

Nature Portfolio wishes to improve the reproducibility of the work that we publish. This form provides structure for consistency and transparency in reporting. For further information on Nature Portfolio policies, see our Editorial Policies and the Editorial Policy Checklist.

Statistics

For all statistical analyses, confirm that the following items are present in the figure legend, table legend, main text, or Methods section.

n/a	Confirmed	
☐	☒	The exact sample size (*n*) for each experimental group/condition, given as a discrete number and unit of measurement
☐	☒	A statement on whether measurements were taken from distinct samples or whether the same sample was measured repeatedly
☐	☒	The statistical test(s) used AND whether they are one- or two-sided<br>*Only common tests should be described solely by name; describe more complex techniques in the Methods section.*
☒	☐	A description of all covariates tested
☒	☐	A description of any assumptions or corrections, such as tests of normality and adjustment for multiple comparisons
☐	☒	A full description of the statistical parameters including central tendency (e.g. means) or other basic estimates (e.g. regression coefficient) AND variation (e.g. standard deviation) or associated estimates of uncertainty (e.g. confidence intervals)
☐	☒	For null hypothesis testing, the test statistic (e.g. *F*, *t*, *r*) with confidence intervals, effect sizes, degrees of freedom and *P* value noted<br>*Give P values as exact values whenever suitable.*
☒	☐	For Bayesian analysis, information on the choice of priors and Markov chain Monte Carlo settings
☒	☐	For hierarchical and complex designs, identification of the appropriate level for tests and full reporting of outcomes
☒	☐	Estimates of effect sizes (e.g. Cohen's *d*, Pearson's *r*), indicating how they were calculated

Our web collection on statistics for biologists contains articles on many of the points above.

Software and code

Policy information about availability of computer code

Data collection	For sensor screening in GSBs and for sensor performance in mammalian cells, fluorescence lifetime images were collected using the ThorImageLS software (v4.2.2020.3061), and thermistor readout was monitored using ThorSync software (v4.0.2019.6171). For acute slice, microscope control and image acquisition were performed by a modified version of the ScanImage software written in MATLAB (Pologruto et al., 2003). For determining the LiLac relative quantum yield, fluorescence measurements were made using a PTI QuantaMaster-8000 spectrofluorometer controlled by PTI Felix 32 software (Photon Technology International).
Data analysis	All FLIM data were managed using laboratory-built software written in MATLAB. Briefly, for sensor screens in GSBs, tiled fluorescence lifetime images are collected for the full coverslip; the first series is analyzed to find all circular ROIs within a selected brightness range (using the stock function imfindcircles.m); and the full image series is analyzed to give dose-response curves for each individual ROI. For experiments in mammalian cells or slices, ROIs were hand-drawn and analyzed. Statistical analyses were performed in Microsoft Excel 2010, GraphPad Prism 7.02, and ImageJ 1.53c.

For manuscripts utilizing custom algorithms or software that are central to the research but not yet described in published literature, software must be made available to editors and reviewers. We strongly encourage code deposition in a community repository (e.g. GitHub). See the Nature Portfolio guidelines for submitting code & software for further information.

Data

Policy information about availability of data

All manuscripts must include a data availability statement. This statement should provide the following information, where applicable:

- Accession codes, unique identifiers, or web links for publicly available datasets
- A description of any restrictions on data availability
- For clinical datasets or third party data, please ensure that the statement adheres to our policy

Source data are provided with this paper.

Field-specific reporting

Please select the one below that is the best fit for your research. If you are not sure, read the appropriate sections before making your selection.

☒ Life sciences ☐ Behavioural & social sciences ☐ Ecological, evolutionary & environmental sciences

For a reference copy of the document with all sections, see nature.com/documents/nr-reporting-summary-flat.pdf

Life sciences study design

All studies must disclose on these points even when the disclosure is negative.

Sample size	Sample size was selected based on sample availability, to give a reasonable estimation of variability.
Data exclusions	No data were excluded.
Replication	All experiments were replicated and results were repeatable. The replication number of each experiment is described in the corresponding figure and/or figure caption.
Randomization	Samples were randomly allocated into groups.
Blinding	Blinding was not required as there were no opportunities for experimenter bias.

Reporting for specific materials, systems and methods

We require information from authors about some types of materials, experimental systems and methods used in many studies. Here, indicate whether each material, system or method listed is relevant to your study. If you are not sure if a list item applies to your research, read the appropriate section before selecting a response.

Materials & experimental systems

n/a	Involved in the study	
☒	☐	Antibodies
☐	☒	Eukaryotic cell lines
☒	☐	Palaeontology and archaeology
☐	☒	Animals and other organisms
☒	☐	Human research participants
☒	☐	Clinical data
☒	☐	Dual use research of concern

Methods

n/a	Involved in the study	
☒	☐	ChIP-seq
☒	☐	Flow cytometry
☒	☐	MRI-based neuroimaging

Eukaryotic cell lines

Policy information about cell lines

Cell line source(s)	HEK293T cells were obtained from ATCC and transiently transfected with sensor DNA. No stable cell lines were constructed.
Authentication	Cells were not authenticated as they come from a reliable source.
Mycoplasma contamination	Cells were not tested for mycoplasma contamination.
Commonly misidentified lines (See ICLAC register)	No commonly misidentified cell lines were used in this study.

Animals and other organisms

Policy information about studies involving animals; ARRIVE guidelines recommended for reporting animal research

Laboratory animals	Male and female wild-type C57BL/6N mice of between 14 and 24 days-old were used in this study
Wild animals	This study did not involve wild animals
Field-collected samples	This study did not involve field-collected samples
Ethics oversight	All experiments were performed in compliance with the NIH Guide for the Care and Use of Laboratory Animals and Animal Welfare Act. Specific protocols were approved by the Harvard Medical Area Standing Committee on Animals.

Note that full information on the approval of the study protocol must also be provided in the manuscript.
